# Effects of a one-day shadowing experience on dental students’ attitudes toward ageing and geriatric dentistry: a prospective controlled cohort study

**DOI:** 10.1186/s12909-026-09317-1

**Published:** 2026-04-29

**Authors:** Anna-Lena Hillebrecht, Luisa Dörr, Nadine Schlueter, Philipp Linde, Daniel R. Reissmann, Benedikt C. Spies, Kirstin Vach, Stefan Rupf

**Affiliations:** 1 Department of Prosthetic Dentistry, Center for Dental Medicine, Faculty of Medicine, Medical Center ‑ University of Freiburg, Hugstetter Str. 55, Freiburg, 79106 Germany; 2 Department of Conservative Dentistry, Periodontology and Preventive Dentistry, Hannover Medical School (MHH), Hannover, Germany; 3 Department of Radiation Oncology, Cyberknife and Radiation Therapy, Faculty of Medicine and University Hospital of Cologne, University of Cologne, Kerpener St 62, Cologne, 50937 Germany; 4 Department of Prosthodontics, University of Leipzig, Leipzig, Germany; 5 Institute of Medical Biometry and Statistics, Faculty of Medicine, University of Freiburg, Stefan-Meier-Str. 26, Freiburg, 79104 Germany; 6 Synoptic Dentistry, Saarland University, Kirrbergerstr. 100, Building 73, Homburg Saar, Homburg, 66421 Germany

**Keywords:** Ageism, Dental education, Geriatric attitude, Geriatric dentistry, Gerodontology, Long-term care, Shadowing experience

## Abstract

**Background:**

Ageism among healthcare professionals can undermine the quality of care for older adults, particularly those with high support needs in long-term care. Undergraduate dental curricula often provide limited exposure to care-dependent older persons, which may reinforce stereotypes and reduce preparedness. This study investigated whether a brief shadowing experience in a long-term care facility could influence dental students’ attitudes toward older adults.

**Methods:**

In this prospective controlled cohort study, third-semester dental students (intervention group, IG) completed a one-day shadowing experience, observing a geriatric dentistry specialist during clinical rounds in a long-term care facility. Second-semester students (control group, CG) had no such exposure. All participants completed the 14-item German Geriatric Attitudes Scale (GAS; range 14–70, higher = more positive) at baseline (T0), immediately post-intervention (T1), and at 3-month follow-up (T2). Additional Likert-type items assessed motivation, career alignment, and perceived preparedness. Changes were analysed using t-tests, Wilcoxon tests, and regression models.

**Results:**

Of 77 consenting students, 63 completed baseline (IG = 29; CG = 34). Baseline GAS scores did not differ between groups (IG: 49.2 ± 4.1; CG: 48.5 ± 7.5; *p* = 0.640). At T2, GAS scores increased significantly in the IG (mean change = + 1.84 ± 4.29; *p* = 0.042; Cohen’s d = 0.40), but not in the CG (mean change = + 0.92 ± 4.00; *p* = 0.250). No effect was observed immediately post-intervention. Further analysis showed that improvements were more pronounced among students who had been less positive at the outset.

**Conclusions:**

A single day of shadowing in long-term care can produce a modest but measurable improvement in dental students’ attitudes toward older adults, particularly among those with initially less positive views. While such brief experiences alone may not increase career interest in geriatric dentistry, embedding them within longitudinal, mentored curricula could contribute to reducing ageism and better preparing graduates to meet the oral health needs of ageing populations.

**Supplementary Information:**

The online version contains supplementary material available at 10.1186/s12909-026-09317-1.

## Introduction

The ageing of the population poses considerable challenges to healthcare systems, including the provision of dental care [1]. While many older adults remain independent in managing their oral health, a growing proportion become dependent on external support, particularly in long-term care settings. These individuals often have complex health needs and rely on caregivers for both daily oral hygiene and access to dental services. Natural teeth or dentures, such as removable prostheses or implant-supported prostheses must be continuously maintained and regularly examined [2–4]. 

Oral health significantly impacts overall health, well-being, and quality of life, especially among older adults with chronic diseases or functional impairments [5–8]. Despite this, dependent older persons continue to face substantial barriers to dental care and consistently exhibit poorer oral health outcomes than the general population [6, 9–11]. 

Addressing this gap requires not only structural changes in dental service delivery but also a dental workforce equipped to meet the evolving needs of the ageing population.

Negative attitudes toward older adults significantly undermine the willingness and commitment to provide appropriate and compassionate care for this population [12]. Ageism contributes to discriminatory practices, insufficient geriatric training in health professions education, and the misattribution of treatable conditions to the normal ageing process [13–18]. The WHO’s Global Oral Health Strategy envisions universal access to oral health by 2030, highlighting the need for equitable, evidence-based, and lifecycle-oriented dental services, including for older adults and residents of long-term care facilities. In alignment with this, the FDI Vision 2030: Delivering Optimal Oral Health for All emphasizes the importance of integrating oral health into general health care and developing a resilient, person-centred dental workforce that is prepared to meet the needs of ageing populations [19, 20]. 

The centrality of gerodontology in dental education is not new. Since the European College of Gerodontology released undergraduate guidelines in 2009, numerous national and international efforts have emphasised the importance of competency-based training in caring for older adults [21]. Nonetheless, many curricula still offer limited, structured exposure to care-dependent populations, which is particularly relevant component for nurturing positive attitudes and professional commitment. Gerodontology training in many dental schools still remains insufficient, with significant gaps in both didactic content and clinical exposure [22–25]. 

While some institutions have incorporated relevant topics, others continue to lack systematic instruction on ageing, frailty, and collaborative care. In Germany, where curriculum design was historically decentralised, the new licensing regulations, which prescribe interdisciplinary gerodontology teaching, represent an important shift [26]. Moreover, the European Commission’s 2022 directive now requires all member states to include gerodontology training in undergraduate dental education [27]. 

To adequately prepare future dentists, curricula need to promote not only knowledge and skills, but also attitudes and motivation to work with older adults, particularly those with care needs [23, 28–31]. Didactic content is important, but insufficient on its own to reduce or prevent ageism, both conscious and unconscious [32–34]. Previous research highlights the importance of clinical exposure in shaping dental students’ attitudes toward older adults. While casual interactions with older individuals in everyday life appear to have limited impact, a higher number of older patients treated during dental training has been associated with more positive attitudes [35]. 

However, dependent older persons, such as those residing in long-term care facilities or requiring assistance for daily activities, are rarely encountered in routine student clinical training. Due to the complexity of their medical conditions and care needs, they are typically not treated in undergraduate dental clinics. As a result, students often lack professional contact to this particularly vulnerable subgroup of the ageing population.

In addition to students, educators play a pivotal role in shaping attitudes towards older adults and in preventing ageism in health care education.Educators who have regular, meaningful clinical contact with older adults tend to exhibit lower levels of ageism, defined as stereotypes, prejudice, and discrimination based on age, which aligns with improved attitudes and demonstrate greater empathy towards ageing patients. These psychosocial shifts are not merely attitudinal: they also appear to increase self-efficacy and willingness to engage with gerodontology, making the integration of relevant content into teaching more likely [36]. 

Although interprofessional, practice-based geriatric training programs have been shown to enhance student competencies and collaboration in caring for older adults [37, 38], there is a lack of evidence regarding the effectiveness of short, discipline-specific observational experiences, particularly in undergraduate dental education, on shaping students’ attitudes towards aging and care-dependent seniors. Observational experiences may offer unique advantages by providing authentic first-hand insights into the realities of geriatric care. Undergraduate training is a critical period for developing professional values, with around two-thirds of dental graduates entering clinical practice immediately after graduation. Therefore, early exposure to older and care-dependent patients may have a disproportionate long-term influence on clinical attitudes and behaviour [39, 40]. Amid crowded curricula, brief, purposeful shadowing is an effective way to develop professionalism and prepare for practice [41]. 

Against this background, the present study aimed to evaluate the impact of a one-day observational experience in a long-term care setting on dental students’ attitudes toward older adults. Conducted in collaboration with specialists in geriatric dentistry, the intervention provided students with the opportunity to observe real-world dental care for older adults with high support needs and limited ability to access a dental office independently. Attitudinal change was assessed using the Geriatric Attitudes Scale (GAS), a validated and widely used instrument in medical, nursing, and dental education [42–45]. 

The null hypothesis was that participation in a one-day shadowing experience in a long-term care facility would not result in a statistically significant difference in dental students’ attitudes toward older adults, as measured by the GAS, compared with students who did not participate.

## Materials and methods

This prospective controlled cohort study involved first and second year dental students at a German university (Freiburg im Breisgau/Baden-Württemberg). Third-semester students comprised the intervention group, while second-semester students, who had not yet completed the shadowing experience, served as the control group. Participation in the study was voluntary.

Students in the intervention group completed a one-day shadowing experience, observing a dentist providing care in a long-term care facility. The experience included witnessing clinical procedures and patient interactions, offering students first-hand insight into the challenges and rewards of geriatric dentistry. Students in the control group completed all study assessments prior to their own subsequent participation in the shadowing program.

### Didactic embedding of the shadowing experience

Before the intervention, all students attended a preparatory lecture in gerodontology addressing demographic trends and oral health challenges in ageing populations. Prior to this, students only received standard preclinical teaching (Cariology, Periodontology, and Preventive Dentistry) and technical propaedeutics (preclinical laboratory/technical skills). These courses did not comprise gerodontology-specific learning objectives, simulated geriatric scenarios, or any clinical contact with older or care-dependent adults. At the time of data collection, the students had not yet started to provide patient care.

During the intervention, students accompanied a dentist specialised in geriatric care during clinical rounds in a long-term care facility. Examinations were performed in residents’ rooms to accommodate limited mobility and cognitive impairments. Each visit included at minimum a structured oral health assessment (caries detection, periodontal screening, prosthetic evaluation) and interactions with patients and nursing staff. In addition to observing, students undertook non-clinical supporting tasks such as handing instruments and completing oral health assessment forms. The intervention was explicitly framed within principles of experiential and situated learning: a pre-visit briefing activated prior knowledge and contextualised expectations, while a post-visit debriefing with the treating dentist fostered reflection, consolidation of observations, and integration into the students’ emerging professional identity.

### Data collection and instruments

Students completed a survey including the German version of the 14-item GAS (Supplementary Material 3), scored on a 5-point Likert scale [46]. Higher scores indicated more positive attitudes (range 14–70). Surveys were distributed at baseline (T0), immediately post-shadowing (T1), and 3-months follow-up (T2).

Demographic data collected included age, gender, prior healthcare training, and previous contact with older adults.

Four additional Likert-type items assessed students’ willingness to treat older adults, the willingness to practice in nursing homes, interest in specializing in gerodontology (i.e., pursuing advanced training or academic focus in dental care for older adults, rather than a formally recognized specialty), and a self-rated confidence interacting with seniors.The first Item “I am highly motivated to pursue further qualifications and advanced training in the field of geriatric dentistry” served to assess student motivation for continued education in this field. The second item, “I envision my future clinical practice as being closely aligned with the provision of comprehensive dental care for dependent older adults”, aimed to evaluate the alignment of students’ envisioned clinical careers with the needs of this patient population. To assess attitudes toward the healthcare setting, we included the item “I envision my future clinical practice as being closely aligned with the provision of comprehensive dental care in long-term care facilities” (Item 3). Self-perceived competence was measured via the item “I feel adequately prepared to address the specific needs and challenges associated with providing dental care to older patients.” (Item 4).Primary outcome was the change in GAS sum score from baseline (T0) to three-month follow-up (T2).

During the three-month follow-up interval, third-semester students had not yet started treating patients. Therefore, any changes in attitude observed during the follow-up period cannot be attributed to direct treatment experience.

### Statistics

No formal sample size calculation was conducted for this study. As no prior data were available to reliably estimate the expected effect size, a conventional power analysis was not feasible. Therefore, all accessible students from the relevant semesters were invited to participate. Given the exploratory character of the study and the aim to include the entire eligible cohort, this approach was considered both pragmatic and appropriate.

Changes in GAS sum scores over time and between groups were evaluated using paired and unpaired t-tests. Linear regression models adjusting for GAS sum score at T0 were used to quantify the effect of single variables age, gender, group and prior healthcare training on the GAS sum score changes. For analyzing single GAS items Wilcoxon rank-sum tests and sign tests were used. The data were analyzed using STATA (version 17.0, College Station, TX, USA) with a significance level of 5%.

GAS sum score distributions and changes are visualized using box-and-whisker plots stratified by group and time point (T0, T1, T2). Additionally, line graphs depict mean GAS sum score trajectories for both the intervention and control groups over time, enabling a visual comparison of attitudinal trends. The four Likert-type items were visualized using arrow plots to illustrate individual changes across time points.

### Ethics approval

The study was approved by the university’s ethics committee (No. 22-1396-S1). All students gave informed consent before being enrolled in the study and participated voluntarily.

## Results

The null hypothesis stated that there would be no significant difference in GAS scores between students who participated in the one-day shadowing experience in a long-term care facility (intervention group) and those who did not (control group). This hypothesis was rejected for the change from baseline (T0) to three-month follow-up (T2): the IG showed a statistically significant improvement in GAS scores (mean change = + 1.84 ± 4.29, *p* = 0.042), whereas the CG did not (mean change = + 0.92 ± 4.00, *p* = 0.250). No significant differences between groups were observed immediately after the intervention (T1).

A total of 77 students (out of 86 eligible) consented to participate in the study. At baseline (T0), 63 students completed the survey: 34 in the control group and 29 in the intervention group. Table 1 presents the survey completion rates across all three time points.

**Table 1:**
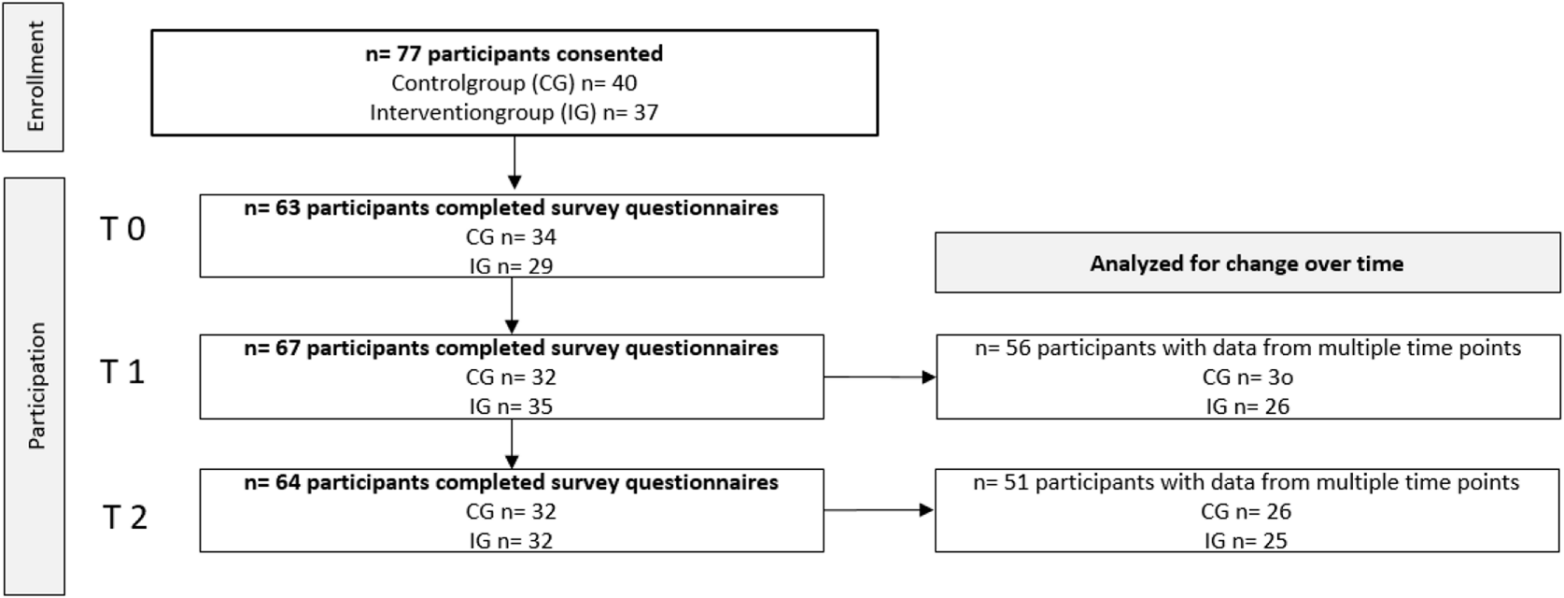
Flow of participants through the study and survey completion rates by group and timepoint

The demographic characteristics of participants did not differ significantly between groups in terms of age, gender, or prior healthcare experience. The majority (87.3%) indicated having regular contact with older adults. The average age was 21.8 ± 2.4 years, and 80.9% (*n* = 51) of participants was identified as female. A prior professional qualification in a healthcare profession (dental nurse, dental technician, paramedic, physiotherapist or nurse) was reported by 47.6% (*n* = 30) of students (Table 2).

**Table 2:** Sociodemographic profile of the intervention and control groups at baseline (T0)

	All	Age	Gender		Prior professional qualification
			Female	Male	
	n	mean (SD)	n (%)	n (%)	n (%)
Intervention group	29	22.2 (2.7)	21 (72%)	8 (28%)	15 (52%)
Control group	34	21.4 (2.2)	30 (88%)	4 (12%)	15 (44%)

### Geriatric attitudes

#### GAS sum score

There was no statistically significant difference in the GAS sum scores at baseline between the intervention group and control group (mean ± SD: 49.2 ± 4.1 vs. 48.5 ± 7.5, *p* = 0.640). Likewise, the baseline attitudes did not significantly differ between groups regarding age (*p* = 0.694), prior qualification (*p* = 0.183), or regular contact with older adults (*p* = 0.287). However, male participants scored lower on the GAS than females (43.8 ± 9.3 vs. 50.0 ± 4.6, *p* = 0.001), but were in the minority with only 19%.

At immediate post-intervention (T1), no statistically significant changes were observed in the GAS sum scores in either group. The intervention group showed a mean increase of 0.96 ± 4.3 points (*p* = 0.261), while the control group changed by 0.61 ± 3.7 points (*p* = 0. 398), indicating no immediate intervention effect.

**Fig. 1:**
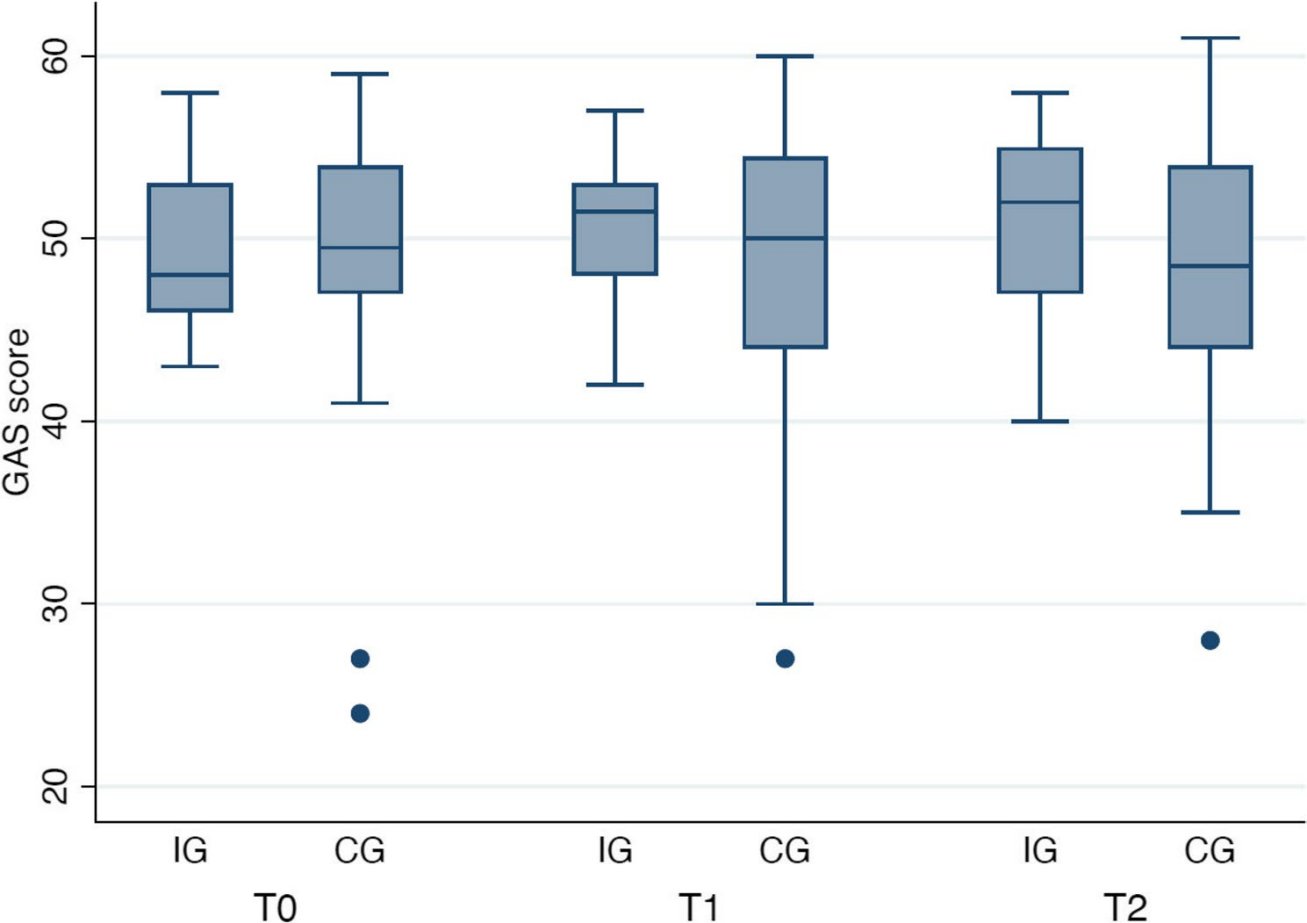
GAS sum score at time points T0, T1, T2 of the Intervention group (IG) and the Control group (CG)

At three-month follow-up (T2), mean geriatric attitude scores in the intervention group were slightly higher than at baseline (49.2 ± 4.4 to 51.0 ± 4.9; mean change = + 1.84, *p* = 0.042). In the control group, scores increased from 47.7 ± 8.3 to 48.7 ± 7.9 (mean change = + 0.92, *p* = 0.250). Figure 1 illustrates the development of mean GAS sum scores over the three measurement points (T0, T1, T2) for both the intervention group and the control group.

#### Cluster-based analysis

To explore patterns of attitudinal change, both the intervention and control groups were stratified post hoc into three clusters based on the change in GAS sum score from baseline (T0) to follow-up (T2):

- *Cluster 0* (Decrease): decrease in GAS score by ≥ 2 points
- *Cluster 1* (Stable): GAS difference between − 2 and + 2
- *Cluster 2* (Increase): increase in GAS score by ≥ 2 points

In the intervention group, participants in Cluster 2 (*n* = 13), who exhibited the most pronounced improvements, had the lowest baseline GAS scores (M = 47.8, SD = 3.8).

**Fig. 2:**
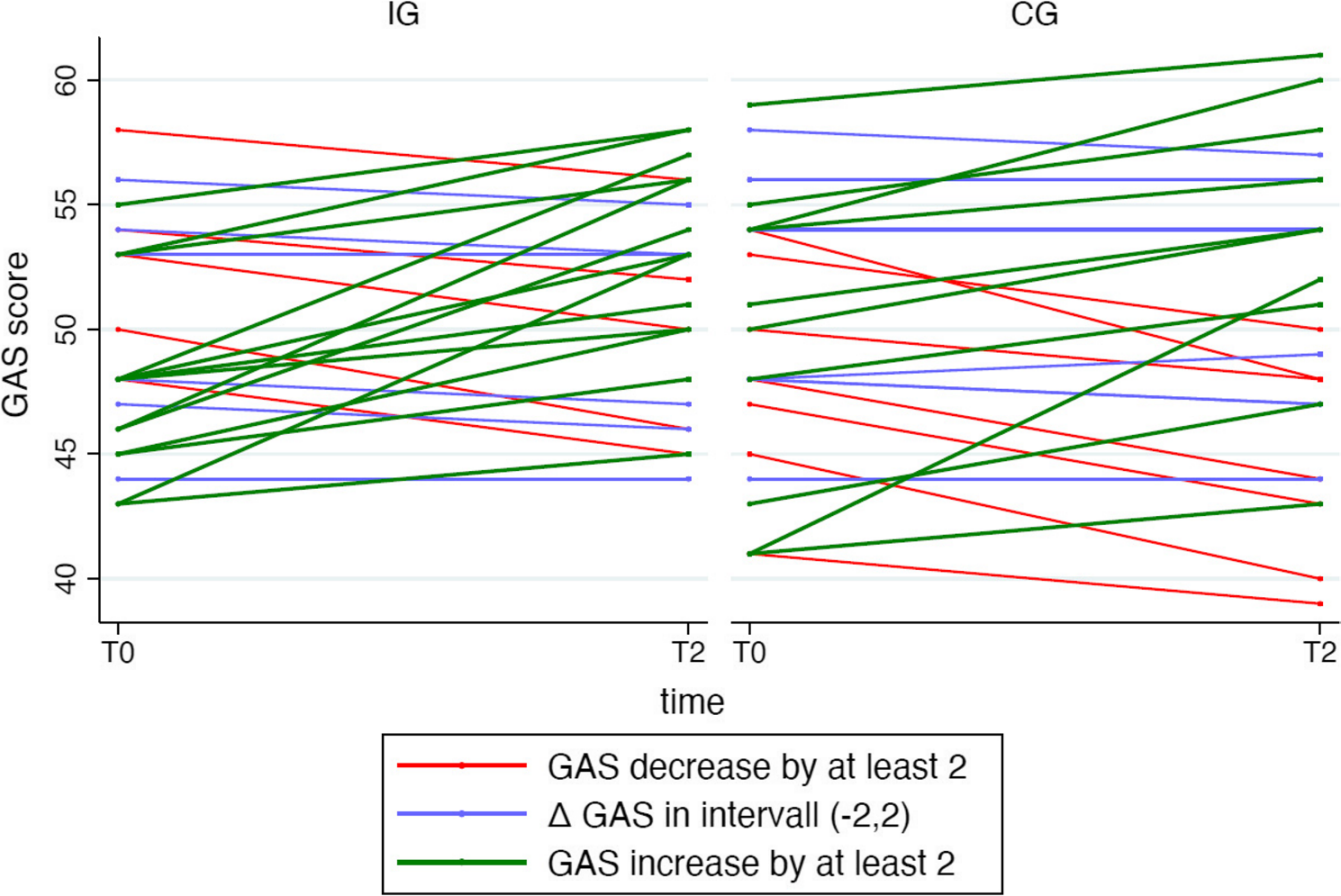
Individual GAS score trajectories over three time points (T0 = baseline, T2 = 3-month follow-up), stratified by group. Each line represents one participant. Two outliers from the control group were removed

Individual trajectories of GAS scores over the three measurement points are illustrated in Fig. 2.

Conversely, those in Cluster 0 (*n* = 5), who showed declining scores, had the highest initial values (M = 52.6, SD = 3.9). A comparable, albeit less distinct, trend was observed in the control group. In the intervention group, there were no notable differences in age across clusters (Cluster 0: M = 22.2 ± 1.8 years; Cluster 1: M = 22.5 ± 3.6; Cluster 2: M = 21.9 ± 1.6). The same applied to the control group, where age also varied only slightly (Cluster 0: M = 22.3 ± 3.1; Cluster 1: M = 22.0 ± 3.0; Cluster 2: M = 21.3 ± 1.2). Thus, no consistent association between age and attitudinal change was observed. Excluding two outliers, which in the CG consistently exhibited low GAS scores, did not materially alter the overall pattern of results.

Regarding gender, the distribution was similarly balanced across clusters in both groups. In the IG, female students made up the majority in all clusters (77% in Cluster 2), as they did in the CG (100% in Cluster 2). No gender-specific pattern was found.

With respect to prior healthcare experience, a slightly higher proportion of students in Cluster 2 (improved attitudes) reported having a vocational background in health professions (IG: 62%; CG: 70%) compared to Clusters 0 and 1. However, this trend was not consistent or statistically robust due to the small subgroup sizes and should be interpreted with caution.

Analysis of regular contact with older adults revealed no meaningful differences across clusters. In both groups, a large majority of students reported regular interactions with older people, regardless of attitudinal trajectory (IG: 83%; CG: 92%).

Multiple linear regression using GAS score change from T0 to T2 as the dependent variable identified prior vocational training as the strongest predictor of attitudinal improvement (regression coefficient = 3.42; *p* = 0.002). Age showed a non-significant positive trend (regression coefficient = 1.25; *p* = 0.268). Due to multicollinearity between age and training, both were not included simultaneously in the final model. Baseline GAS score was a significant negative predictor of change, indicating that students with lower initial attitudes benefited most.

#### GAS items-level analysis

To gain deeper insight into specific dimensions of students’ attitudinal change, individual items of the GAS were analysed separately. Within the intervention group, a statistically significant improvement from T0 to T1 was observed for Item 2 (“The statutory health insurance funds should invest the money for dental treatment of senior citizens in the treatment of younger risk groups.“) and Item 12 (“Old people do not contribute a fair share to paying for their healthcare.“).

While the control group showed a significant increase in agreement with Item 5 (“Medical care for the elderly consumes too many human and material resources.“), this effect was not mirrored in the IG. Intergroup comparisons at individual time points revealed significant differences between the control group and intervention group for Items 2 and 12 at T1 and T2 reflecting group-specific intervention effects (Table-Supplement).

#### Exploratory assessment of motivation, professional orientation, and perceived preparedness

**Fig. 3:**
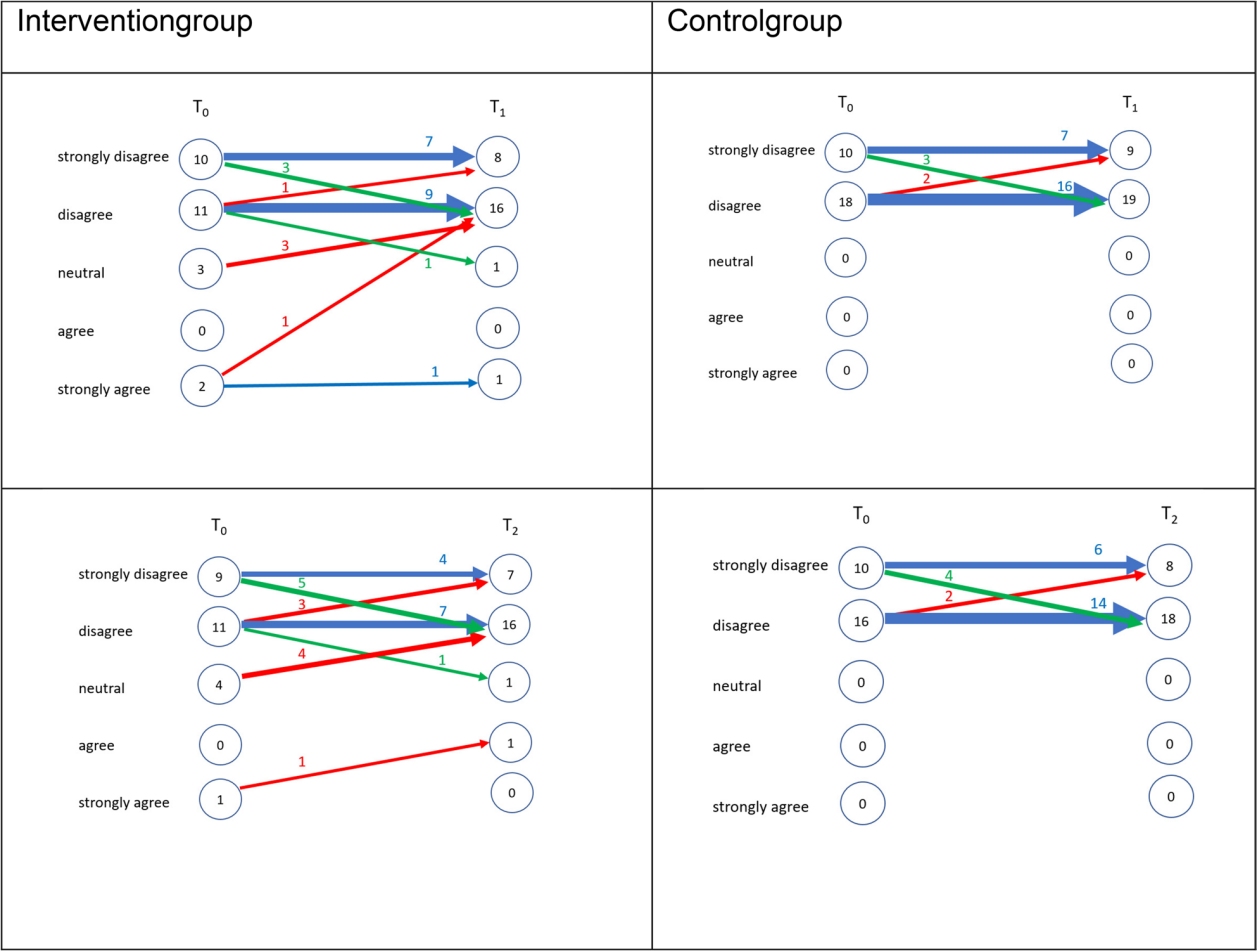
Motivation to pursue further qualifications in geriatric dentistry from baseline (T0) to post-intervention (T1) and follow-up (T2) in the intervention and control groups. Item: “I am highly motivated to pursue further qualifications and advanced training in geriatric dentistry.” Each line represents one participant. Red denotes a decrease, blue no change, and green an increase

**Fig. 4:**
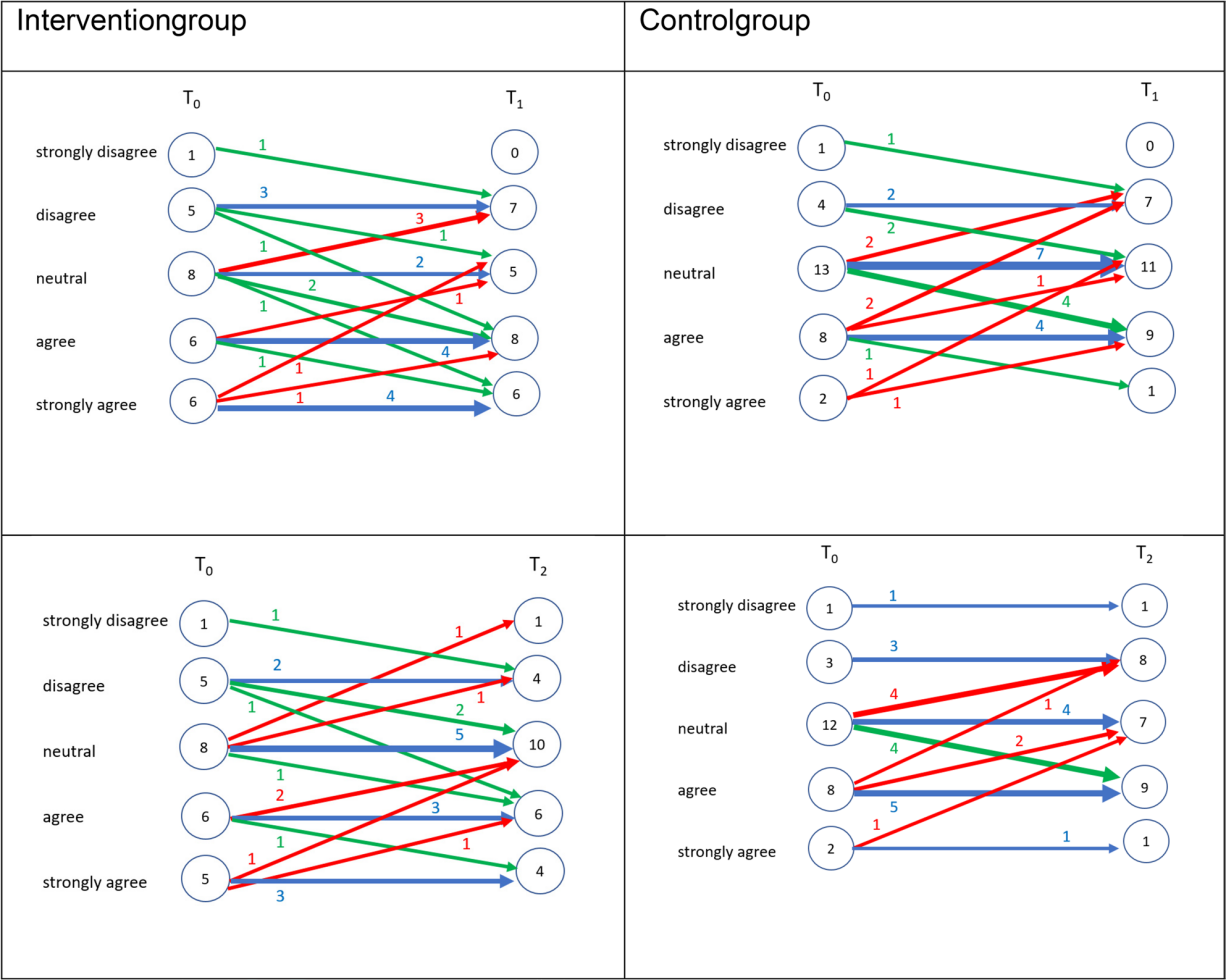
Alignment of envisioned future clinical practice with the care of dependent older adults from baseline (T0) to post-intervention (T1) and follow-up (T2) in the intervention and control groups. Item: “I envision my future clinical practice as being closely aligned with the provision of comprehensive dental care for dependent older adults.” Each line represents one participant. Red denotes a decrease, blue no change, and green an increase

**Fig. 5:**
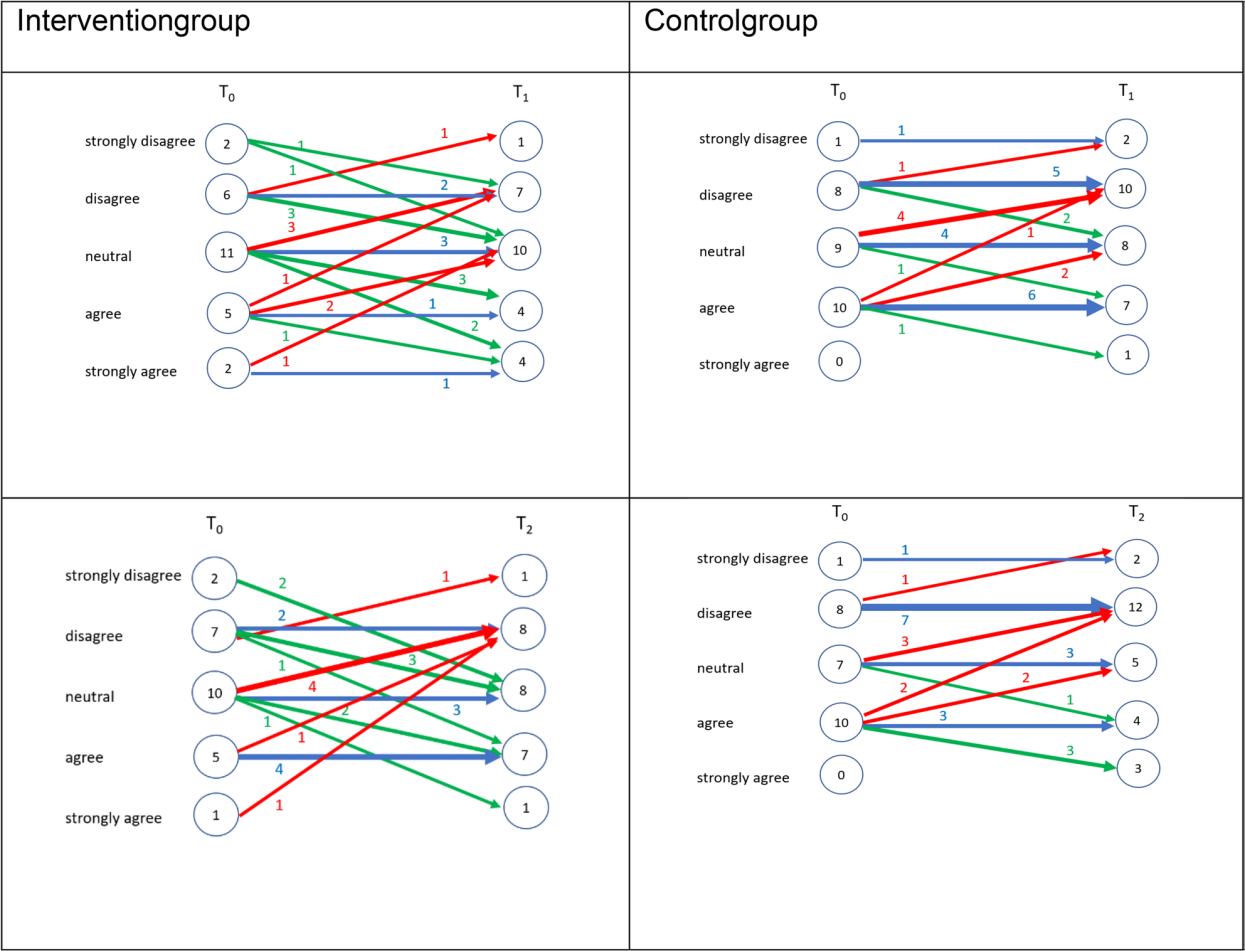
Perceived alignment of envisioned future clinical practice with dental care provision in long-term care facilities from baseline (T0) to post-intervention (T1) and follow-up (T2) in the intervention and control groups. Item: “I envision my future clinical practice as being closely aligned with the provision of comprehensive dental care in long-term care facilities.” Each line represents one participant. Red denotes a decrease, blue no change, and green an increase

**Fig. 6:**
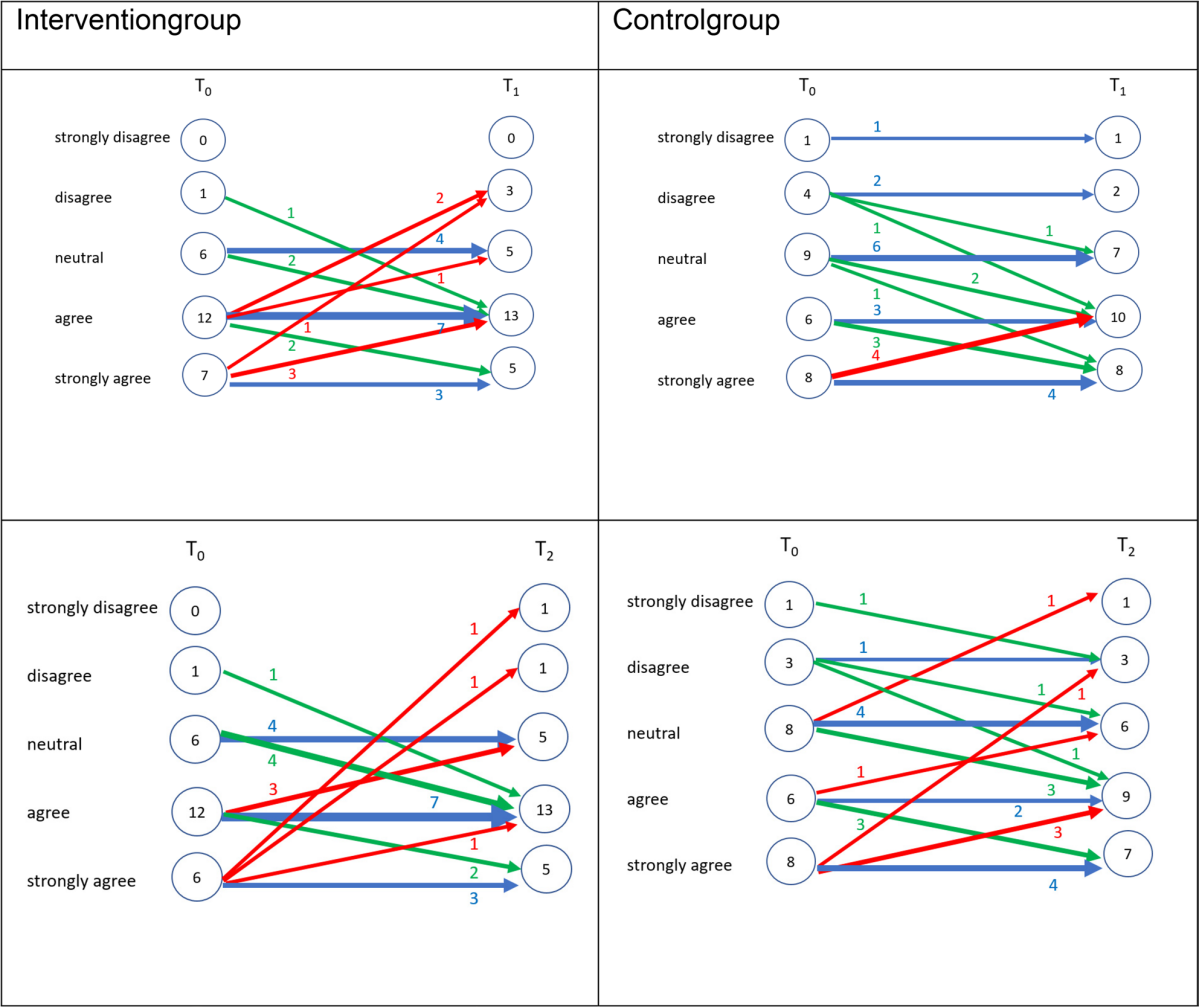
Self-perceived preparedness to address the needs of older dental patients from baseline (T0) to post-intervention (T1) and follow-up (T2) in the intervention and control groups. Item: “I feel adequately prepared to address the specific needs and challenges associated with providing dental care to older patients.” Each line represents one participant. Red denotes a decrease, blue no change, and green an increase

Four additional Likert-type items assessed students’ motivation for further training, perceived career alignment with geriatric dentistry, preference for long-term care-based care, and self-assessed preparedness. Mean values remained relatively stable and low across time, with a slight decline from T0 to T2 observed for all items. (T1), followed by a return to baseline levels at T2. The highest mean score across all time points was observed for perceived preparedness (mean = 3.78, SD = 0.99), while professional alignment remained low (mean = 1.79, SD = 0.73). Figures 3, 4, 5 and 6 illustrate the individual response trajectories for the four exploratory items.

## Discussion

### Main findings and significance

This study investigated the potential of a brief, real-world shadowing experience in long-term care to enhance dental students’ attitudes toward older adults. The results demonstrate a small improvement in Geriatric Attitudes Scale (GAS) scores three months post-intervention. This finding prompts consideration of how small effect sizes should be interpreted in the context of attitudinal change. Although the observed effect size in this study was small (Cohen’s d = 0.4), this should not be interpreted as pedagogically insignificant. In educational contexts targeting complex constructs such as empathy or attitudinal change, even small improvements may indicate meaningful developmental progress. As Kraft emphasizes, even small-to-moderate changes can lead to substantial long-term outcomes, especially when embedded early in professional identity formation [47]. This interpretation is further supported by a pooled effect size of d = 0.42 (95% CI: 0.01–0.83) reported by Başer and Hisar across 13 studies targeting attitudes toward older adults in nursing and medical students, which closely aligns with our findings [48]. 

However, the exploratory items on motivation, career orientation, and preparedness remained low and largely stable, indicating that attitudinal change may not directly translate into professional orientation or career-related motivation.

### Mechanisms of attitude change

Attitudes are multifaceted constructs comprising cognitive, affective, and behavioural components, and can be conceptualized along the tripartite model of attitudes [49]. Educational interventions targeting attitude change must therefore address three domains: factual knowledge (cognitive), emotional responses (affective), and behavioural intention (behavioural), to be effective. Shadowing experiences in long-term care settings confront learners with the real-life implications of aging on oral health (cognitive), elicit emotional responses to residents’ dependence (affective), and demonstrate how to address such needs professionally as a dentist, which may shape future professional intentions (behavioural). The quality of contact, rather than its duration, may be key to promoting sustained attitudinal change. Mihevc et al., emphasize the educator’s role in addressing ageism in health professions education [36]. Witnessing best-practice approaches in geriatric dental care (including respectful communication, patient-centred decision-making, and interdisciplinary teamwork) can serve as powerful role modelling experiences. Positive role models in clinical settings not only demonstrate professional skills but also implicitly communicate values, attitudes, and norms that students may integrate into their own professional identity. Embedding such shadowing opportunities into the curriculum, ideally through repeated and mentored experiences, could thus enhance the development of age-sensitive, reflective practitioners. This is in line with Kolb’s experiential learning theory, which frames learning as a cyclical process involving concrete experience, reflective observation, abstract conceptualization, and active experimentation. Shadowing care-dependent older adults provides a meaningful “concrete experience” within this cycle, while structured reflection and follow-up discussions facilitate the transition to deeper learning phases [50]. Recent work in health professions education has emphasized that attitude change is not merely a function of knowledge acquisition, but deeply rooted in individual dispositions, personality traits, and lived experiences. Personality characteristics such as openness to experience, agreeableness, and empathy have been associated with more positive attitudes toward older adults and greater readiness to engage in care for dependent populations [51]. Conversely, traits like age anxiety, or death anxiety may foster more negative perceptions and resistance to attitudinal change [15]. 

### Delayed effects and learner variability

No significant change was observed immediately after the shadowing experience (T1). This delayed effect may reflect ongoing internalisation and perspective shifts that occur beyond the immediate learning context. Evidence from nursing and simulation-based education suggests that repeated clinical exposures significantly enhance learners’ self-confidence, clinical competence, and critical thinking skills [52]. Individual trajectories revealed that some students experienced a decline in interest or perceived alignment with geriatric dentistry. Such ambivalent or negative responses may reflect a realistic confrontation with the emotional and practical complexities of caring for dependent older adults. Rather than viewing these reactions as pedagogical shortcomings, they should be considered meaningful triggers for critical self-reflection. To harness this potential and prevent disengagement, interventions should be accompanied by structured debriefing, psychological safety, and longitudinal mentorship.

Notably, students with lower baseline GAS scores benefited most from the intervention, suggesting that those with less positive initial views may be particularly responsive to reflective, real-world learning experiences. This pattern aligns with Transformative Learning Theory, which posits that disorienting experiences (when adequately supported) can challenge existing frames of reference and catalyse profound perspective shifts [53]. Students’ career alignment with geriatric dentistry remained low across time points, and some reported decreased interest after the intervention. This may reflect the emotionally demanding nature of long-term care and the limited visibility of geriatric dentistry as a professional pathway. For future iterations, both the preparatory briefing and the debriefing should be optimised and sufficiently supported to ensure that all students are well prepared for, and can critically reflect on, the encounter, thereby maximising its positive impact. Incorporating brief reflective writing or guided discussion assignments could further reinforce the affective learning component further and has been shown to make attitude changes more durable.

### Comparison with other educational approaches

The overall GAS scores in our study clustered around a total value of 50 on a scale from 14 to 70, indicating a generally positive, though not yet optimal, attitudinal profile. This level of agreement is consistent with previously reported values: for instance, a GAS mean of 51.8 was observed among U.S. medical students [54], and similar findings were reported among Swiss dental students (≈ 49) [46] and dental hygiene students [55]. These converging data points suggest that, across disciplines and countries, health professions students tend to hold moderately positive views toward older adults, yet al.so underscore the need for targeted interventions to further strengthen age-sensitive professional attitudes.

The post-intervention GAS mean in our study 3.48 (SD = 0.56) was somewhat lower than the 3.82 (SD = 0.43) reported following a semester-long gerodontology course in Brazil [56]. The results are also consistent with findings from another study reporting significant attitudinal improvements after interdisciplinary, simulation-based geriatric training [57]. While these more intensive interventions may produce larger absolute gains, their implementation requires considerably greater curricular resources and scheduling flexibility. Compared to simulation-based or didactic formats, observational shadowing provides an authentic, emotionally salient, and low-threshold entry point into geriatric care, allowing dental students to witness real patient–provider encounters, foster empathy, and challenge ageist stereotypes, thereby priming them for deeper competency-based learning.

### Implications for curriculum design

The results of this study support the growing international consensus that targeted educational interventions are a key strategy in counteracting ageism in health care. A recent systematic review and meta-analysis demonstrated that interventions aimed at reducing ageist attitudes, especially those combining educational elements with intergenerational contact, significantly improve attitudes, knowledge, and comfort in dealing with older adults [32]. These strategies are particularly relevant given epidemiological data from the Fifth German Oral Health Study (DMS 5), which highlight the growing oral health needs of care-dependent older persons [58]. The shadowing experience alone may be insufficient to substantially enhance students’ motivation, career interest, or preparedness in geriatric dentistry, but can serve as an entry point into longitudinal gerodontology training. Integrating gerodontology shadowing experience early in the curriculum may raise awareness of complex geriatric needs and provide authentic exposure to patient populations rarely seen in dental school clinics.

### Limitations

This study has several limitations that should be considered when interpreting the findings. The sample size was relatively small, which may limit generalizability and statistical power. Participation was voluntary, introducing potential selection bias. As data were self-reported, responses may have been influenced by social desirability. The absence of follow-up beyond three months limits the ability to draw conclusions about the sustainability of observed changes. A further limitation is that the debriefing was not tailored to individual learners’ needs; future implementations should adopt a more person-centred approach to facilitate deeper reflection and address diverse emotional and cognitive responses. As both groups received the same introductory lecture and standard preclinical courses, some of the observed change may be due to general maturation or an increased awareness resulting from the concurrent teaching, rather than the shadowing experience alone. Future studies should more explicitly isolate these effects.

## Conclusion

A single day of shadowing in long-term care can lead to a measurable, albeit modest, improvement in dental students’ attitudes toward older adults. When embedded in a longitudinal, mentored framework, such experiences can contribute to reducing ageism in dentistry and preparing graduates to meet the oral health needs of aging populations. Further research is needed to validate and expand these findings.

## Supplementary Information

Supplementary Material 1: Table-Supplement: Mean values ± standard deviations of GAS-Score items and sum GAS-Score for the timepoints T0, T1 and T2 and difference between T0/T1, and T0/T2 within the intervention (IG)- and control group (CG).

Supplementary Material 2: Figure-Supplement: Distribution of GAS score changes from post-intervention to follow-up (T1–T0 and T2-T0) in intervention group (IG) and control group (CG).

Supplementary Material 3: GAS- Questionnaire.

## Data Availability

The datasets generated and/or analysed during the current study are not publicly available due to data protection regulations but are available from the corresponding author on reasonable request.

## Abbreviations

CG: Control group
FDI: FDI World Dental Federation
GAS: Geriatric Attitudes Scale
IG: Intervention group
SD: Standard deviation
WHO: World Health Organization
