# Supplementary material for: Effects of a one-day shadowing experience on dental students’ attitudes toward ageing and geriatric dentistry: a prospective controlled cohort study
Source: BMC Med Educ. 2026 Apr 29;26:694. doi: 10.1186/s12909-026-09317-1 (PMC13126811; doi:10.1186/s12909-026-09317-1)
Supplement: Supplementary file 1 — Supplementary Material 1: Table-Supplement: Mean values ± standard deviations of GAS-Score items and sum GAS-Score for the timepoints T0, T1 and T2 and difference between T0/T1, and T0/T2 within the intervention (IG)- and control group (CG). [file 12909_2026_9317_MOESM1_ESM.docx]

**Table S1.** Mean values ± standard deviations of GAS item scores and total GAS scores at time points T0, T1, and T2, and changes from T0 to T1 and from T0 to T2 in the intervention group (IG) and control group (CG). *Significant within-group changes.* Within each column, significant differences between the control group and intervention group for individual items are indicated by different superscript letters.

| Item | Group | T0 | T1 | T2 | Δ (T1-T0) | Δ (T2-T0) |
| --- | --- | --- | --- | --- | --- | --- |
| GAS 1 | CG | 3.71 ± 0.87^a^ | 3.63 ± 0.93^a^ | 3.77 ± 0.95^a^ | -0.03 ±0.72^a^ | 0.08 ± 0.69^a^ |
|  | IG | 3.66 ± 0.86^a^ | 3.46 ± 0.65^a^ | 3.72 ± 0.79^a^ | -0.23 ± 0.70^a^ | 0.12 ± 0.60^a^ |
| GAS 2 | CG | 3.56 ± 0.93^a^ | 3.50 ± 1.04^a^ | 3.69 ± 0.93^a^ | -0.18 ± 1.02^a^ | 0.00 ± 0.80^a^ |
|  | IG | 3.59 ± 0.95^a^ | 3.92 ± 0.69^a^ | 3.84 ± 0.85^a^ | **0.39 ± 0.85^b^** | 0.20 ± 1.00^a^ |
| GAS 3 | CG | 2.53 ± 1.02^a^ | 2.57 ± 0.96^a^ | 2.92 ± 0.85^a^ | -0.04 ± 0.79^a^ | 0.27 ± 0.67^a^ |
|  | IG | 2.79 ± 1.21^a^ | 2.50 ± 1.21^a^ | 2.88 ± 0.93^a^ | -0.23 ± 1.03^a^ | 0.20 ± 1.08^a^ |
| GAS 4 | CG | 4.15 ± 0.86^a^ | 4.18 ± 0.98^a^ | 4.19 ± 0.85^a^ | 0.04 ± 1.00^a^ | 0.04 ± 0.66^a^ |
|  | IG | 4.41 ± 0.63^a^ | 4.42 ± 0.64^a^ | 4.28 ± 0.61^a^ | -0.04 ± 0.60^a^ | -0.16 ± 0.47^a^ |
| GAS 5 | CG | 3.27 ± 1.08^a^ | 3.71 ± 1.08^a^ | 3.58 ± 1.07^a^ | **0.50 ± 0.92*^a^** | **0.42 ± 0.90*^a^** |
|  | IG | 3.76 ± 0.95^a^ | 3.65 ± 1.16^a^ | 3.96 ± 0.84^a^ | **-0.19 ±1.23^b^** | 0.08 ± 0.91^a^ |
| GAS 6 | CG | 2.59 ± 0.78^a^ | 2.61 ± 0.88^a^ | 2.62 ± 0.75^a^ | 0.04 ± 0.79^a^ | 0.08 ± 0.74^a^ |
|  | IG | 2.38 ± 0.62^a^ | 2.54 ± 1.00^a^ | 2.64 ± 0.64^a^ | 0.12 ± 1.00^a^ | 0.24 ± 0.66^a^ |
| GAS 7 | CG | 3.21 ± 0.98^a^ | 3.07 ± 0.90^a^ | 3.08 ± 1.06^a^ | -0.11 ± 0.83^a^ | -0.04 ± 0.92^a^ |
|  | IG | 2.97 ± 0.68^a^ | 3.00 ± 0.90^a^ | 3.20 ± 0.77^a^ | 0.00 ± 0.94^a^ | 0.24 ± 0.72^a^ |
| GAS 8 | CG | 3.50 ± 0.86^a^ | 3.50 ± 0.92^a^ | 3.50 ± 1.00^a^ | -0.04 ± 1.04^a^ | -0.15 ± 1.22^a^ |
|  | IG | 3.62 ± 0.94^a^ | 3.62 ± 0.75^a^ | 3.44 ± 0.90^a^ | -0.08 ± 1.09^a^ | -0.16 ± 0.94^a^ |
| GAS 9 | CG | 2.47 ± 0.96^a^ | 2.46 ± 1.23^a^ | 2.46 ± 1.33^a^ | 0.36 ± 0.75^a^ | 0.04 ± 1.00^a^ |
|  | IG | 2.24 ± 0.95^a^ | 2.50 ± 1.14^a^ | 2.48 ± 1.05^a^ | 0.27 ± 1.19^a^ | 0.28 ± 1.00^a^ |
| GAS 10 | CG | 4.15 ± 1.05^a^ | 4.00 ± 1.05^a^ | 4.04 ± 0.82^a^ | -0.07 ± 0.54^a^ | 0.04 ± 0.92^a^ |
|  | IG | 3.86 ± 0.79^a^ | 4.08 ± 1.00^a^ | 4.20 ± 0.76^a^ | 0.15 ± 1.05^a^ | 0.24 ± 0.83^a^ |
| GAS 11 | CG | 4.15 ± 1.08^a^ | 4.11 ± 1.03^a^ | 3.92 ± 1.06^a^ | 0.11 ± 1.00^a^ | 0.00 ± 0.75^a^ |
|  | IG | 4.38 ± 0.78^a^ | 4.39 ± 0.80^a^ | 4.36 ± 0.76^a^ | -0.04 ± 1.00^a^ | 0.04 ± 0.74^a^ |
| GAS 12 | CG | 3.79 ± 0.95^a^ | 3.89 ± 1.00^a^ | 3.85 ± 0.83^a^ | 0.18 ± 0.91^a^ | 0.19 ± 1.10^a^ |
|  | IG | 4.04 ± 0.73^a^ | **4.50 ± 0.58^b^** | **4.36 ± 0.64^b^** | **0.46 ± 0.86*^a^** | 0.32 ± 1.03^a^ |
| GAS 13 | CG | 3.35 ± 1.10^a^ | 3.18 ± 0.91^a^ | 3.12 ± 0.91^a^ | -0.07 ± 0.72^a^ | -0.08 ± 0.69^a^ |
|  | IG | 3.28 ± 0.80^a^ | 3.54 ± 0.76^a^ | 3.44 ± 0.92^a^ | 0.27 ± 1.12^a^ | 0.12 ± 1.17^a^ |
| GAS 14 | CG | 4.06 ± 1.01^a^ | 4.25 ± 0.84^a^ | 4.08 ± 0.98^a^ | 0.25 ± 0.84^a^ | 0.04 ± 0.72^a^ |
|  | IG | 4.24 ± 0.87^a^ | 4.31 ± 0.79^a^ | 4.24 ± 0.72^a^ | 0.12 ± 1.14^a^ | 0.08 ± 1.12^a^ |
| GAS SUM | CG | 48.81 ± 7.52^a^ | 48.64 ± 8.12^a^ | 48.65 ± 7.87^a^ | 0.61 ± 3.74^a^ | 0.92 ± 4.00^a^ |
|  | IG | 49.21 ± 4.13^a^ | 50.42 ± 4.01^a^ | 51.04 ± 4.88^a^ | 0.96 ± 4.27^a^ | **1.84 ± 4.29*^a^** |
| GAS Item∅ | CG | 3.46 ± 0.54 | 3.48 ± 0.58 | 3.48 ± 0.56 | 0.04 ± 0.27 | **0.07 ± 0.29** |
|  | IG | 3.51 ± 0.29 | 3.60 ± 0.29 | 3.65 ± 0.35 | 0.07± 0.31 | **0.13 ± 0.31** |
