# Supplementary material for: Effects of a one-day shadowing experience on dental students’ attitudes toward ageing and geriatric dentistry: a prospective controlled cohort study
Source: BMC Med Educ. 2026 Apr 29;26:694. doi: 10.1186/s12909-026-09317-1 (PMC13126811; doi:10.1186/s12909-026-09317-1)
Supplement: Supplementary file 2 — Supplementary Material 2: Figure-Supplement: Distribution of GAS score changes from post-intervention to follow-up (T1–T0 and T2-T0) in intervention group (IG) and control group (CG). [file 12909_2026_9317_MOESM2_ESM.docx]

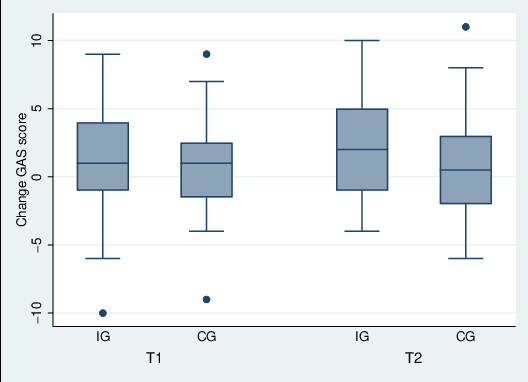


**Figure-Supplement 1:** Distribution of GAS score changes from post-intervention to follow-up (T1–T0 and T2-T0) in intervention group (IG) and control group (CG)


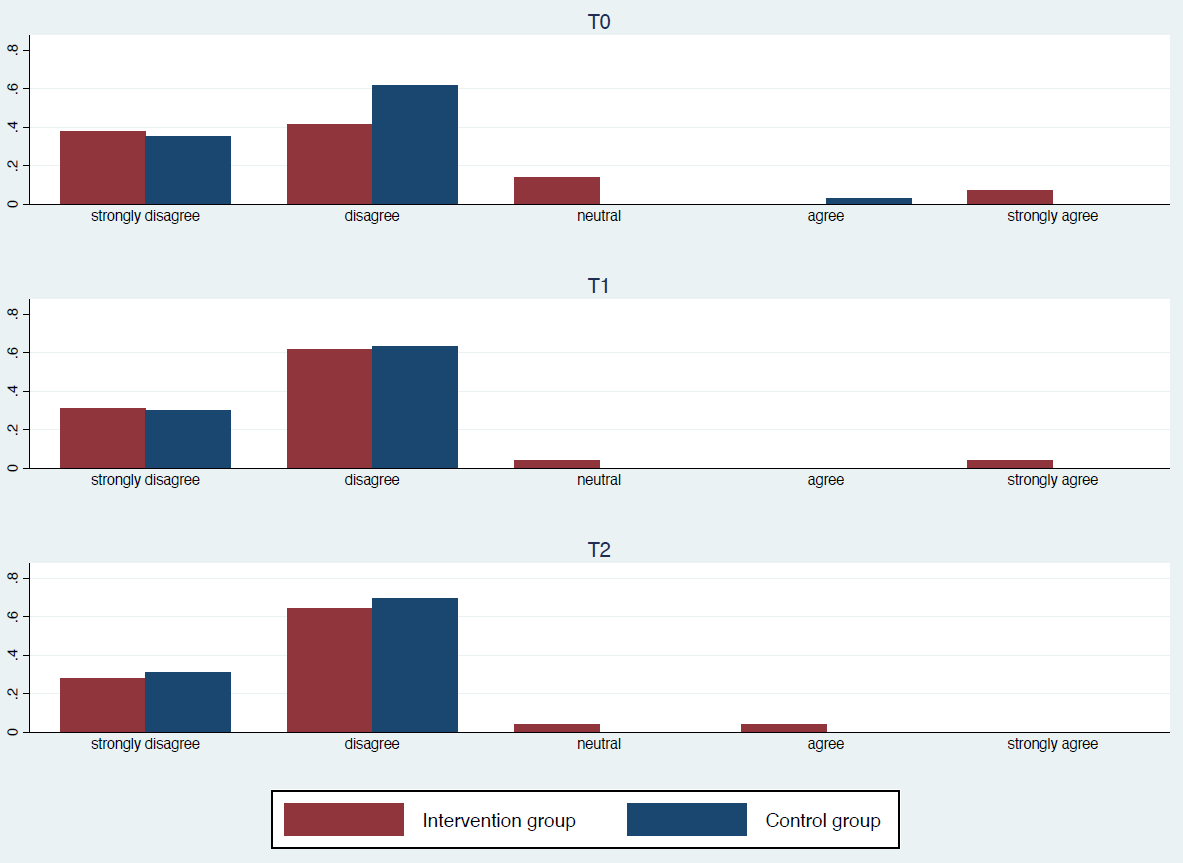


**Figure-Supplement 2:**  “I am highly motivated to pursue further qualifications and advanced training in the field of geriatric dentistry”. Motivation to pursue further qualifications in geriatric dentistry (Item 1).


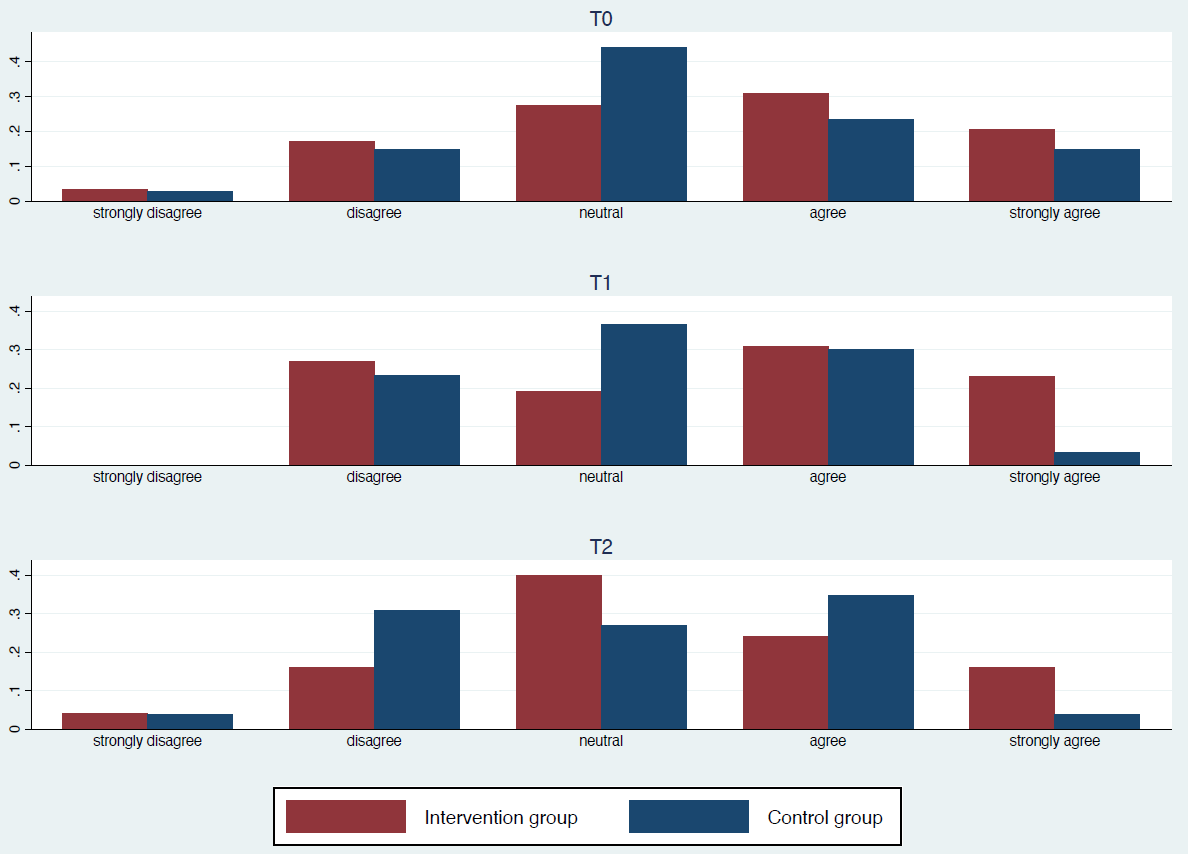


**Figure-Supplement 3:** “I envision my future clinical practice as being closely aligned with the provision of comprehensive dental care for dependent older adults”. Alignment of future clinical practice with the care of dependent older adults (Item 2).


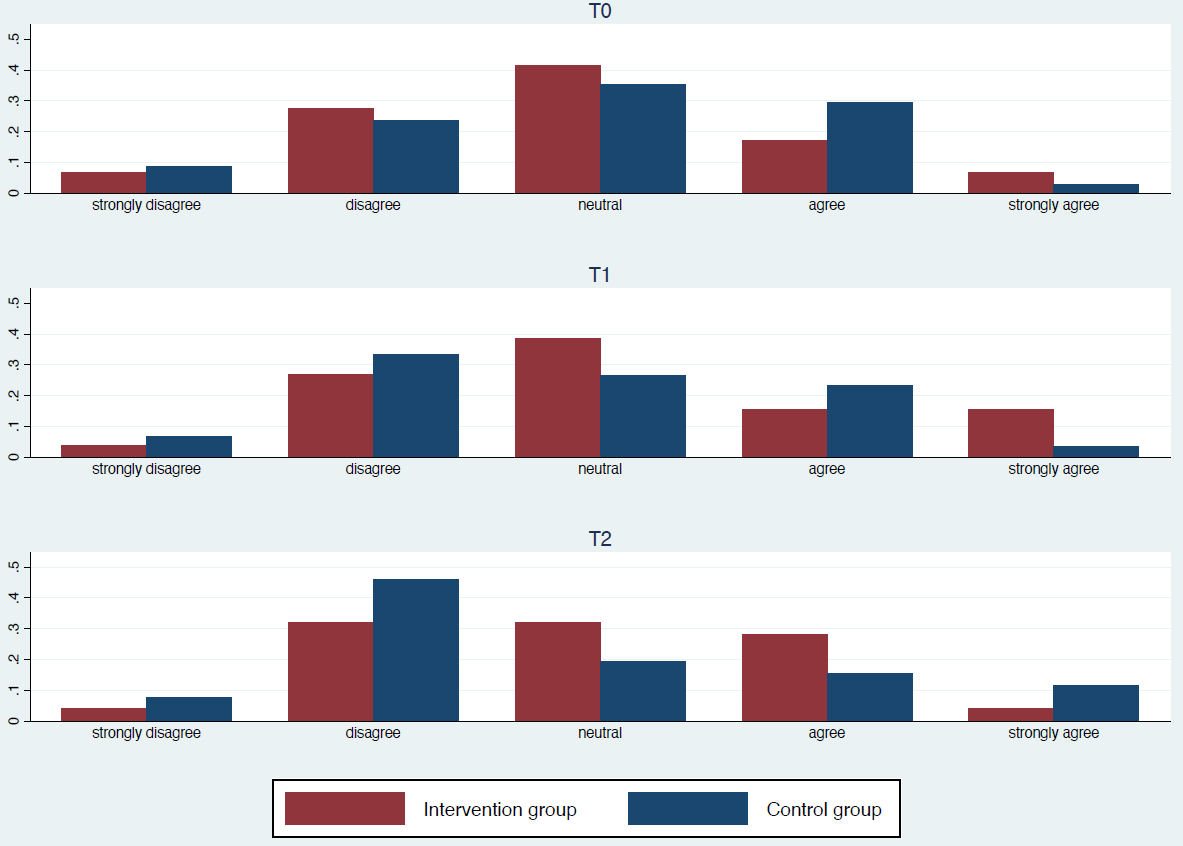


**Figure-Supplement 4:** “I envision my future clinical practice as being closely aligned with the provision of comprehensive dental care in long-term care facilities”. Perceived dental care services in long-term care facilities (Item 3).


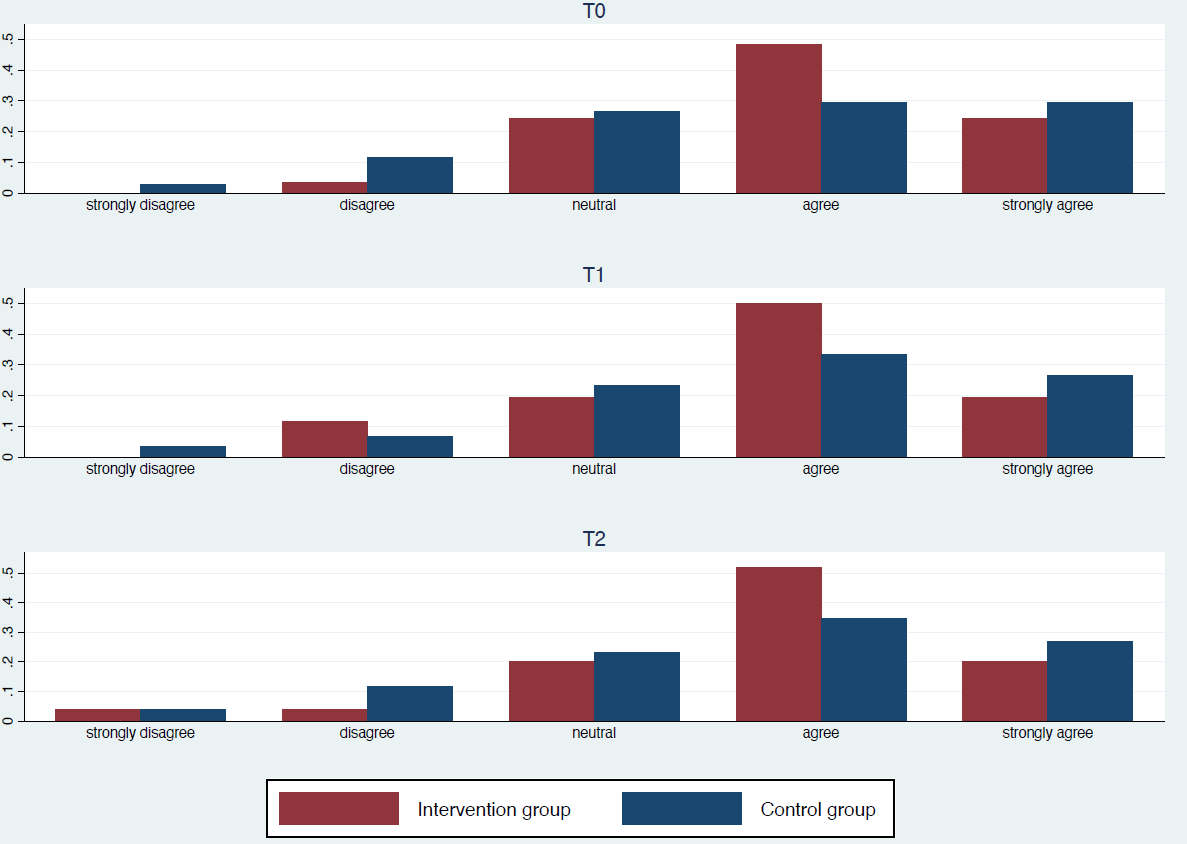


**Figure-Supplement 5:** “I feel adequately prepared to address the specific needs and challenges associated with providing dental care to older patients”. Self-perceived preparedness to address the needs of older dental patients (Item 4).
