# Supplementary material for: Effects of a one-day shadowing experience on dental students’ attitudes toward ageing and geriatric dentistry: a prospective controlled cohort study
Source: BMC Med Educ. 2026 Apr 29;26:694. doi: 10.1186/s12909-026-09317-1 (PMC13126811; doi:10.1186/s12909-026-09317-1)
Supplement: Supplementary file 3 — Supplementary Material 3: GAS- Questionnaire. [file 12909_2026_9317_MOESM3_ESM.docx]

**German version of the Geratric Attitudes Scale**

ANWEISUNGEN: Bitte geben Sie auf der Skala an, inwieweit Sie den einzelnen Aussagen zustimmen oder nicht zustimmen. Es gibt keine richtigen oder falschen Antworten. Die beste Antwort ist die, die Ihre persönliche Meinung widerspiegelt. Die Ergebnisse dieser Studie werden nur auf Gruppenbasis und ohne Nennung von Einzelpersonen berichtet. „Alte Menschen“ und ‚ältere Patienten‘, die in den Fragen erwähnt werden, beziehen sich auf Personen, die 65 Jahre oder älter sind.

1. Mit den meisten älteren Leuten bin ich gerne zusammen.

2. Die gesetzlichen Krankenkassen sollten das Geld für die zahnmedizinische Behandlung von Senior*innen eher in die Behandlung von jüngeren Risikogruppen investieren.

3. Wenn ich die Wahl hätte, würde ich lieber jüngere als alte Patient*innen behandeln.

4. Eine Gesellschaft ist dafür verantwortlich, sich um ihre älteren Personen zu kümmern.

5. Die medizinische Versorgung alter Menschen verbraucht zu viele personelle und materielle Ressourcen.

6. Wenn Menschen älter werden, werden sie weniger organisiert und verwirrter.

7. Ältere Patient*innen haben tendenziell eine höhere Wertschätzung für die medizinische Versorgung, die ich anbiete, als jüngere Patient*innen.

8. Eine Krankengeschichte von älteren Patient*innen zu erheben ist häufig eine Qual.

9. Ich neige dazu, älteren Personen mehr Aufmerksamkeit und Sympathie entgegenzubringen als jüngeren Personen.

10. Alte Menschen tragen im Allgemeinen nicht viel zur Gesellschaft bei.

11. Die Behandlung chronisch kranker alter Patient*innen ist hoffnungslos.

12. Alte Menschen tragen keinen gerechten Anteil zur Bezahlung ihrer Gesundheitsversorgung bei.

13. Im Allgemeinen handeln alte Menschen für die moderne Gesellschaft zu langsam.

14. Es ist interessant, den Berichten alter Menschen über ihre früheren Erfahrungen zuzuhören.

**Geratric Attitudes Scale**

DIRECTIONS: Please use the scale to indicate the degree to which you agree or disagree with each statement. There are no right or wrong answers. The best response is the one that truly reflects your personal opinion. Findings of this study will be reported only on a group basis with no individual names identified. “Old people” and “elderly patients” mentioned in the questions refer to persons aged 65 or older.

1. I enjoy being with most older people.

2. the statutory health insurance funds should invest the money for dental treatment of senior citizens in the treatment of younger risk groups.

3 If I had the choice, I would rather treat younger patients than older ones.

4. a society has a responsibility to look after its older people

5 Medical care for the elderly consumes too many human and material resources.

6. as people get older, they become less organised and more confused

7. older patients tend to have a higher appreciation for the medical care I provide than younger patients.

8. taking a medical history from older patients is often a pain.

9. i tend to be more attentive and sympathetic to older people than younger people.

10. old people generally do not contribute much to society.

11. the treatment of chronically ill elderly patients is hopeless.

12. old people do not contribute a fair share to paying for their healthcare.

13. in general, old people act too slowly for modern society

14. it is interesting to listen to old people talk about their past experiences.
